# Bis[5-meth­oxy-2-(meth­oxy­carbon­yl)phen­yl] methyl­phospho­nate

**DOI:** 10.1107/S1600536814002542

**Published:** 2014-02-12

**Authors:** Saghir Hussain, Yang Deli, Shagufta Parveen, Xin Hao, Changjin Zhu

**Affiliations:** a School of Chemical Engineering and Environment, Beijing Institute of Technology, Beijing 100081, People’s Republic of China

## Abstract

In the title phospho­nate, C_19_H_21_O_9_P, the dihedral angle between the benzene rings is 63.33 (3)°, and the P atom has a distorted tetra­hedral geometry, with angles in the range 101.30 (6)–120.38 (6)°. No significant inter­molecular inter­actions are observed in the crystal structure, and π–π inter­actions between symmetry-related benzene rings are beyond 4 Å.

## Related literature   

For amidation and esterification of phospho­ric acid, see: Kasemsuknimit *et al.* (2011 ▶). For the biological activity of phospho­nic acids and their ester derivatives, see: Hilderbrand & Henderson (1983 ▶); Das *et al.* (2009 ▶); Wang *et al.* (2012 ▶).
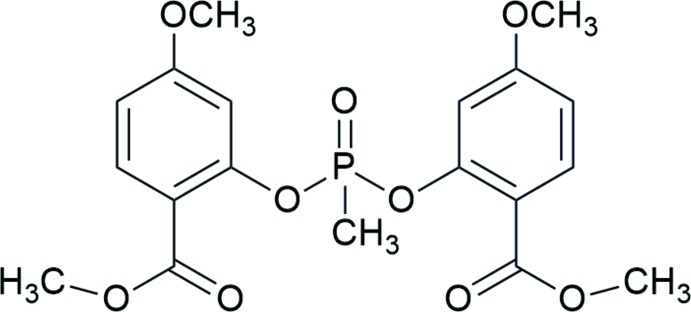


## Experimental   

### 

#### Crystal data  

C_19_H_21_O_9_P
*M* *_r_* = 424.33
Triclinic, 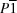

*a* = 7.852 (2) Å
*b* = 10.616 (3) Å
*c* = 12.200 (3) Å
α = 70.774 (9)°
β = 77.800 (9)°
γ = 84.172 (10)°
*V* = 938.0 (4) Å^3^
*Z* = 2
Mo *K*α radiation
μ = 0.20 mm^−1^
*T* = 153 K
0.60 × 0.32 × 0.23 mm

#### Data collection  

Rigaku AFC10/Saturn724+ diffractometer
Absorption correction: multi-scan (*CrystalClear*; Rigaku, 2008 ▶) *T*_min_ = 0.890, *T*_max_ = 0.955
13574 measured reflections
5875 independent reflections
5141 reflections with *I* > 2σ(*I*)
*R*_int_ = 0.028

#### Refinement  

*R*[*F* ^2^ > 2σ(*F* ^2^)] = 0.040
*wR*(*F* ^2^) = 0.102
*S* = 1.00
5875 reflections
268 parameters
H-atom parameters constrained
Δρ_max_ = 0.38 e Å^−3^
Δρ_min_ = −0.50 e Å^−3^

### 

Data collection: *CrystalClear* (Rigaku, 2008 ▶); cell refinement: *CrystalClear*; data reduction: *CrystalClear*; program(s) used to solve structure: *SHELXS97* (Sheldrick, 2008 ▶); program(s) used to refine structure: *SHELXL97* (Sheldrick, 2008 ▶); molecular graphics: *CrystalStructure* (Rigaku, 2008 ▶) and *DIAMOND* (Brandenburg, 1999 ▶); software used to prepare material for publication: *CrystalStructure* (Rigaku, 2008 ▶).

## Supplementary Material

Crystal structure: contains datablock(s) I. DOI: 10.1107/S1600536814002542/bh2491sup1.cif

Structure factors: contains datablock(s) I. DOI: 10.1107/S1600536814002542/bh2491Isup2.hkl

Supporting information file. DOI: 10.1107/S1600536814002542/bh2491Isup3.cml

CCDC reference:

Additional supporting information:  crystallographic information; 3D view; checkCIF report

## Figures and Tables

**Fig. 1.**
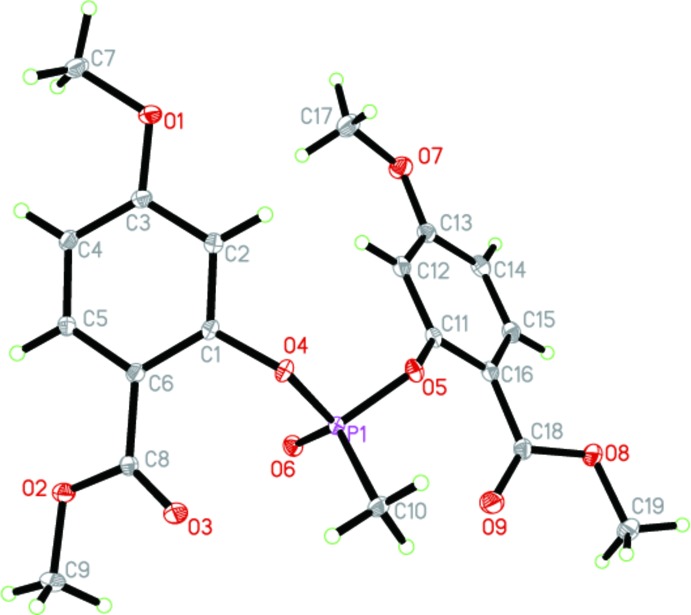
The molecular structure of the title compound with displacement ellipsoids drawn at the 30% probability level.

**Fig. 2.**
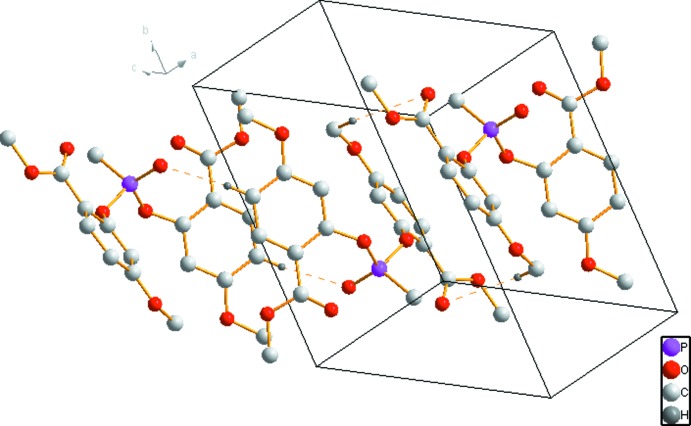
A view of intermolecular interactions (dotted lines) in the crystal structure of the title compound. H atoms not involved in contacts were omitted for clarity.

## supplementary crystallographic information

### 1. Comment

Phosphonic acids are very important in understanding and modulating some biological processes (Hilderbrand & Henderson, 1983). For instance, aminophosphonate *N*-derivatives are potent and selective inhibitors of protein tyrosine phosphatases (Wang *et al.*, 2012). Within this field, phosphonic esters and their derivatives have attracted interest due to their biological activities (Das *et al.*, 2009; Kasemsuknimit *et al.*, 2011). As an extension of these studies, we report herein on the structure of the title compound, C_19_H_21_O_9_P.

The molecule (Fig. 1) is lying in general position in a triclinic cell. The molecular conformation is centered on a distorted tetrahedral P atom, and benzene rings of oxo substituents make a dihedral angle of 63.33 (3)°. In the crystal structure (Fig. 2), very weak C—H···O intermolecular contacts exist, which involve P═O and C═O groups as acceptors.

### 2. Experimental

A mixture of diethyl methylphosphite (10 mmol) and phosphoryl chloride (10.5 mmol) was refluxed at 55 °C for 48 h. Then, 4-methoxy-methylsalicylate (10 mmol) in CH_2_Cl_2_ (10 ml) was added dropwise in the presence of triethylamine (4 mmol) to the viscous yellowish reaction mixture and stirred for 30 minutes at 0 °C, and then at room temperature for 48 h. The reaction mixture was extracted with CH_2_Cl_2_ (3×10 ml) and H_2_O (10 ml). The organic layer was washed with 1*M* HCl (3×10 ml), H_2_O (10 ml), dried over MgSO_4_, concentrated, and the residue was purified by column chromatography with petroleum ether and ethyl acetate (3:1) as eluent, affording a colorless solid.

### 3. Refinement

All H atoms were included in the riding model approximation with C—H distances of 0.95 (aromatic CH) or 0.98 Å (methyl CH_3_), and with *U*_iso_(H)=1.2*U*_eq_(C aromatic) or 1.5*U*_eq_(C methyl).

### Crystal data

**Table:**

C_19_H_21_O_9_P	*Z* = 2
*M**_r_* = 424.33	*F*(000) = 444
Triclinic, *P*1	*D*_x_ = 1.502 Mg m^−^^3^
Hall symbol: -P 1	Mo *K*α radiation, λ = 0.71073 Å
*a* = 7.852 (2) Å	Cell parameters from 3814 reflections
*b* = 10.616 (3) Å	θ = 2.3–31.5°
*c* = 12.200 (3) Å	µ = 0.20 mm^−^^1^
α = 70.774 (9)°	*T* = 153 K
β = 77.800 (9)°	Prism, colorless
γ = 84.172 (10)°	0.60 × 0.32 × 0.23 mm
*V* = 938.0 (4) Å^3^	

### Data collection

**Table:**

Rigaku AFC10/Saturn724+ diffractometer	5875 independent reflections
Radiation source: Rotating Anode	5141 reflections with *I* > 2σ(*I*)
Graphite monochromator	*R*_int_ = 0.028
Detector resolution: 28.5714 pixels mm^-1^	θ_max_ = 31.0°, θ_min_ = 2.3°
φ and ω scans	*h* = −11→11
Absorption correction: multi-scan (*CrystalClear*; Rigaku, 2008)	*k* = −15→14
*T*_min_ = 0.890, *T*_max_ = 0.955	*l* = −17→16
13574 measured reflections	

### Refinement

**Table:**

Refinement on *F*^2^	Secondary atom site location: difference Fourier map
Least-squares matrix: full	Hydrogen site location: inferred from neighbouring sites
*R*[*F*^2^ > 2σ(*F*^2^)] = 0.040	H-atom parameters constrained
*wR*(*F*^2^) = 0.102	*w* = 1/[σ^2^(*F*_o_^2^) + (0.0463*P*)^2^ + 0.36*P*] where *P* = (*F*_o_^2^ + 2*F*_c_^2^)/3
*S* = 1.00	(Δ/σ)_max_ = 0.001
5875 reflections	Δρ_max_ = 0.38 e Å^−^^3^
268 parameters	Δρ_min_ = −0.50 e Å^−^^3^
0 restraints	Extinction correction: *SHELXL97* (Sheldrick, 2008), Fc^*^=kFc[1+0.001xFc^2^λ^3^/sin(2θ)]^-1/4^
0 constraints	Extinction coefficient: 0.018 (2)
Primary atom site location: structure-invariant direct methods	

### Fractional atomic coordinates and isotropic or equivalent isotropic displacement parameters (Å^2^)

**Table:**

	*x*	*y*	*z*	*U*_iso_*/*U*_eq_	
P1	0.33809 (4)	0.27109 (3)	0.77167 (3)	0.01429 (8)	
O1	0.32051 (13)	0.73969 (9)	0.92979 (9)	0.0254 (2)	
O2	0.05122 (13)	0.15477 (9)	1.15552 (9)	0.0245 (2)	
O3	0.29370 (14)	0.10828 (9)	1.03853 (9)	0.0278 (2)	
O4	0.42225 (11)	0.33306 (9)	0.85008 (8)	0.01764 (17)	
O5	0.40476 (11)	0.36743 (9)	0.64092 (8)	0.01864 (18)	
O6	0.14807 (11)	0.26036 (9)	0.80413 (8)	0.01992 (18)	
O7	0.12590 (14)	0.79574 (10)	0.48656 (9)	0.0291 (2)	
O8	0.33993 (14)	0.27659 (10)	0.35051 (9)	0.0279 (2)	
O9	0.32347 (16)	0.17592 (10)	0.54559 (9)	0.0318 (2)	
C1	0.33063 (15)	0.40112 (12)	0.92554 (10)	0.0156 (2)	
C2	0.36082 (15)	0.53541 (12)	0.89291 (11)	0.0180 (2)	
H2	0.4363	0.5776	0.8207	0.022*	
C3	0.27920 (15)	0.60907 (12)	0.96717 (11)	0.0177 (2)	
C4	0.16437 (16)	0.54847 (12)	1.07129 (11)	0.0183 (2)	
H4	0.1058	0.5987	1.1206	0.022*	
C5	0.13718 (16)	0.41317 (12)	1.10167 (11)	0.0184 (2)	
H5	0.0588	0.3718	1.1728	0.022*	
C6	0.22035 (15)	0.33527 (12)	1.03192 (10)	0.0165 (2)	
C7	0.24992 (19)	0.81449 (14)	1.00919 (13)	0.0268 (3)	
H7A	0.1223	0.8166	1.0222	0.032*	
H7B	0.2911	0.9059	0.9747	0.032*	
H7C	0.2882	0.7720	1.0848	0.032*	
C8	0.19625 (16)	0.18952 (12)	1.07215 (11)	0.0182 (2)	
C9	0.0165 (2)	0.01386 (14)	1.19956 (14)	0.0306 (3)	
H9A	0.0128	−0.0178	1.1332	0.037*	
H9B	−0.0959	−0.0006	1.2544	0.037*	
H9C	0.1090	−0.0355	1.2409	0.037*	
C10	0.47462 (17)	0.12702 (12)	0.77410 (12)	0.0216 (2)	
H10A	0.4780	0.0735	0.8562	0.026*	
H10B	0.5927	0.1540	0.7321	0.026*	
H10C	0.4286	0.0739	0.7354	0.026*	
C11	0.30478 (14)	0.45119 (12)	0.56325 (10)	0.0167 (2)	
C12	0.25839 (15)	0.57749 (13)	0.57246 (11)	0.0192 (2)	
H12	0.2823	0.6012	0.6361	0.023*	
C13	0.17609 (16)	0.66892 (13)	0.48675 (12)	0.0210 (2)	
C14	0.14194 (16)	0.63410 (14)	0.39278 (12)	0.0225 (2)	
H14	0.0855	0.6965	0.3346	0.027*	
C15	0.19083 (16)	0.50850 (13)	0.38535 (11)	0.0215 (2)	
H15	0.1691	0.4857	0.3206	0.026*	
C16	0.27195 (15)	0.41295 (13)	0.47087 (11)	0.0186 (2)	
C17	0.1721 (2)	0.83997 (16)	0.57463 (14)	0.0350 (3)	
H17A	0.1159	0.7847	0.6532	0.042*	
H17B	0.1331	0.9334	0.5626	0.042*	
H17C	0.2989	0.8321	0.5686	0.042*	
C18	0.31427 (16)	0.27628 (13)	0.46351 (11)	0.0210 (2)	
C19	0.3690 (2)	0.14795 (15)	0.33229 (14)	0.0308 (3)	
H19A	0.4530	0.0945	0.3797	0.037*	
H19B	0.4152	0.1601	0.2484	0.037*	
H19C	0.2586	0.1019	0.3564	0.037*	

### Atomic displacement parameters (Å^2^)

**Table:**

	*U*^11^	*U*^22^	*U*^33^	*U*^12^	*U*^13^	*U*^23^
P1	0.01521 (13)	0.01433 (14)	0.01354 (15)	−0.00011 (10)	−0.00176 (10)	−0.00540 (11)
O1	0.0306 (5)	0.0164 (4)	0.0281 (5)	−0.0045 (4)	0.0032 (4)	−0.0099 (4)
O2	0.0277 (5)	0.0176 (4)	0.0259 (5)	−0.0049 (3)	0.0029 (4)	−0.0075 (4)
O3	0.0359 (5)	0.0169 (4)	0.0263 (5)	0.0033 (4)	0.0007 (4)	−0.0067 (4)
O4	0.0172 (4)	0.0215 (4)	0.0173 (4)	0.0015 (3)	−0.0030 (3)	−0.0111 (3)
O5	0.0155 (4)	0.0235 (4)	0.0140 (4)	0.0004 (3)	−0.0028 (3)	−0.0024 (3)
O6	0.0168 (4)	0.0226 (4)	0.0200 (4)	−0.0034 (3)	−0.0009 (3)	−0.0069 (4)
O7	0.0344 (5)	0.0224 (5)	0.0298 (5)	0.0054 (4)	−0.0085 (4)	−0.0078 (4)
O8	0.0411 (6)	0.0256 (5)	0.0178 (5)	−0.0018 (4)	−0.0029 (4)	−0.0096 (4)
O9	0.0496 (6)	0.0235 (5)	0.0222 (5)	−0.0008 (4)	−0.0092 (5)	−0.0058 (4)
C1	0.0159 (5)	0.0181 (5)	0.0150 (5)	0.0022 (4)	−0.0041 (4)	−0.0081 (4)
C2	0.0190 (5)	0.0185 (5)	0.0167 (5)	−0.0012 (4)	−0.0027 (4)	−0.0061 (4)
C3	0.0197 (5)	0.0149 (5)	0.0195 (6)	−0.0002 (4)	−0.0048 (4)	−0.0063 (4)
C4	0.0212 (5)	0.0177 (5)	0.0175 (6)	0.0014 (4)	−0.0033 (4)	−0.0083 (4)
C5	0.0213 (5)	0.0182 (5)	0.0150 (5)	−0.0001 (4)	−0.0019 (4)	−0.0055 (4)
C6	0.0188 (5)	0.0157 (5)	0.0155 (5)	0.0009 (4)	−0.0042 (4)	−0.0055 (4)
C7	0.0300 (6)	0.0203 (6)	0.0336 (7)	−0.0014 (5)	−0.0027 (6)	−0.0153 (6)
C8	0.0231 (5)	0.0178 (5)	0.0144 (5)	−0.0004 (4)	−0.0051 (4)	−0.0049 (4)
C9	0.0386 (8)	0.0192 (6)	0.0310 (7)	−0.0095 (5)	0.0006 (6)	−0.0059 (5)
C10	0.0265 (6)	0.0170 (5)	0.0211 (6)	0.0040 (4)	−0.0037 (5)	−0.0076 (5)
C11	0.0140 (4)	0.0206 (5)	0.0130 (5)	−0.0015 (4)	−0.0016 (4)	−0.0022 (4)
C12	0.0175 (5)	0.0229 (6)	0.0168 (6)	−0.0021 (4)	−0.0017 (4)	−0.0064 (5)
C13	0.0179 (5)	0.0208 (6)	0.0213 (6)	−0.0007 (4)	−0.0010 (4)	−0.0045 (5)
C14	0.0201 (5)	0.0256 (6)	0.0187 (6)	−0.0006 (5)	−0.0057 (4)	−0.0016 (5)
C15	0.0208 (5)	0.0266 (6)	0.0165 (6)	−0.0038 (5)	−0.0045 (4)	−0.0046 (5)
C16	0.0173 (5)	0.0220 (6)	0.0157 (5)	−0.0030 (4)	−0.0013 (4)	−0.0053 (5)
C17	0.0497 (9)	0.0257 (7)	0.0301 (8)	0.0022 (6)	−0.0056 (7)	−0.0118 (6)
C18	0.0215 (5)	0.0248 (6)	0.0174 (6)	−0.0031 (4)	−0.0023 (4)	−0.0077 (5)
C19	0.0414 (8)	0.0285 (7)	0.0275 (7)	−0.0033 (6)	−0.0056 (6)	−0.0153 (6)

### Geometric parameters (Å, º)

**Table:**

P1—O6	1.4667 (10)	C7—H7A	0.9800
P1—O5	1.5956 (10)	C7—H7B	0.9800
P1—O4	1.5984 (9)	C7—H7C	0.9800
P1—C10	1.7720 (13)	C9—H9A	0.9800
O1—C3	1.3596 (15)	C9—H9B	0.9800
O1—C7	1.4375 (16)	C9—H9C	0.9800
O2—C8	1.3476 (15)	C10—H10A	0.9800
O2—C9	1.4465 (16)	C10—H10B	0.9800
O3—C8	1.2099 (15)	C10—H10C	0.9800
O4—C1	1.3927 (14)	C11—C12	1.3878 (17)
O5—C11	1.3865 (14)	C11—C16	1.3953 (17)
O7—C13	1.3633 (16)	C12—C13	1.3939 (18)
O7—C17	1.4275 (19)	C12—H12	0.9500
O8—C18	1.3494 (16)	C13—C14	1.3971 (19)
O8—C19	1.4428 (17)	C14—C15	1.3764 (19)
O9—C18	1.2063 (17)	C14—H14	0.9500
C1—C2	1.3791 (17)	C15—C16	1.4057 (18)
C1—C6	1.4048 (17)	C15—H15	0.9500
C2—C3	1.4009 (17)	C16—C18	1.4829 (18)
C2—H2	0.9500	C17—H17A	0.9800
C3—C4	1.3914 (17)	C17—H17B	0.9800
C4—C5	1.3872 (17)	C17—H17C	0.9800
C4—H4	0.9500	C19—H19A	0.9800
C5—C6	1.3977 (16)	C19—H19B	0.9800
C5—H5	0.9500	C19—H19C	0.9800
C6—C8	1.4801 (17)		
			
O6—P1—O5	113.96 (5)	O2—C9—H9C	109.5
O6—P1—O4	114.87 (5)	H9A—C9—H9C	109.5
O5—P1—O4	102.31 (5)	H9B—C9—H9C	109.5
O6—P1—C10	120.38 (6)	P1—C10—H10A	109.5
O5—P1—C10	101.53 (6)	P1—C10—H10B	109.5
O4—P1—C10	101.30 (6)	H10A—C10—H10B	109.5
C3—O1—C7	116.45 (10)	P1—C10—H10C	109.5
C8—O2—C9	115.46 (10)	H10A—C10—H10C	109.5
C1—O4—P1	125.45 (8)	H10B—C10—H10C	109.5
C11—O5—P1	127.58 (8)	O5—C11—C12	117.91 (11)
C13—O7—C17	117.67 (11)	O5—C11—C16	119.54 (11)
C18—O8—C19	116.33 (11)	C12—C11—C16	122.11 (11)
C2—C1—O4	116.28 (10)	C11—C12—C13	118.90 (12)
C2—C1—C6	121.84 (11)	C11—C12—H12	120.6
O4—C1—C6	121.81 (11)	C13—C12—H12	120.6
C1—C2—C3	119.46 (11)	O7—C13—C12	123.89 (12)
C1—C2—H2	120.3	O7—C13—C14	115.62 (12)
C3—C2—H2	120.3	C12—C13—C14	120.48 (12)
O1—C3—C4	124.13 (11)	C15—C14—C13	119.34 (12)
O1—C3—C2	115.46 (11)	C15—C14—H14	120.3
C4—C3—C2	120.41 (11)	C13—C14—H14	120.3
C5—C4—C3	118.65 (11)	C14—C15—C16	121.86 (12)
C5—C4—H4	120.7	C14—C15—H15	119.1
C3—C4—H4	120.7	C16—C15—H15	119.1
C4—C5—C6	122.68 (11)	C11—C16—C15	117.30 (12)
C4—C5—H5	118.7	C11—C16—C18	122.28 (11)
C6—C5—H5	118.7	C15—C16—C18	120.39 (11)
C5—C6—C1	116.89 (11)	O7—C17—H17A	109.5
C5—C6—C8	120.60 (11)	O7—C17—H17B	109.5
C1—C6—C8	122.47 (10)	H17A—C17—H17B	109.5
O1—C7—H7A	109.5	O7—C17—H17C	109.5
O1—C7—H7B	109.5	H17A—C17—H17C	109.5
H7A—C7—H7B	109.5	H17B—C17—H17C	109.5
O1—C7—H7C	109.5	O9—C18—O8	122.93 (12)
H7A—C7—H7C	109.5	O9—C18—C16	126.15 (12)
H7B—C7—H7C	109.5	O8—C18—C16	110.91 (11)
O3—C8—O2	122.46 (12)	O8—C19—H19A	109.5
O3—C8—C6	125.90 (12)	O8—C19—H19B	109.5
O2—C8—C6	111.62 (10)	H19A—C19—H19B	109.5
O2—C9—H9A	109.5	O8—C19—H19C	109.5
O2—C9—H9B	109.5	H19A—C19—H19C	109.5
H9A—C9—H9B	109.5	H19B—C19—H19C	109.5
			
O6—P1—O4—C1	−14.43 (11)	C1—C6—C8—O3	19.6 (2)
O5—P1—O4—C1	109.57 (10)	C5—C6—C8—O2	20.16 (16)
C10—P1—O4—C1	−145.81 (10)	C1—C6—C8—O2	−162.06 (11)
O6—P1—O5—C11	8.28 (12)	P1—O5—C11—C12	85.25 (13)
O4—P1—O5—C11	−116.33 (10)	P1—O5—C11—C16	−102.25 (12)
C10—P1—O5—C11	139.24 (10)	O5—C11—C12—C13	172.42 (10)
P1—O4—C1—C2	−112.42 (11)	C16—C11—C12—C13	0.12 (18)
P1—O4—C1—C6	70.66 (14)	C17—O7—C13—C12	4.73 (19)
O4—C1—C2—C3	−177.17 (10)	C17—O7—C13—C14	−174.46 (12)
C6—C1—C2—C3	−0.26 (18)	C11—C12—C13—O7	−179.55 (11)
C7—O1—C3—C4	5.11 (18)	C11—C12—C13—C14	−0.39 (18)
C7—O1—C3—C2	−174.95 (11)	O7—C13—C14—C15	179.04 (11)
C1—C2—C3—O1	178.09 (11)	C12—C13—C14—C15	−0.18 (19)
C1—C2—C3—C4	−1.97 (18)	C13—C14—C15—C16	1.06 (19)
O1—C3—C4—C5	−177.96 (12)	O5—C11—C16—C15	−171.47 (10)
C2—C3—C4—C5	2.11 (18)	C12—C11—C16—C15	0.71 (17)
C3—C4—C5—C6	−0.03 (19)	O5—C11—C16—C18	10.56 (17)
C4—C5—C6—C1	−2.08 (18)	C12—C11—C16—C18	−177.27 (11)
C4—C5—C6—C8	175.82 (11)	C14—C15—C16—C11	−1.30 (18)
C2—C1—C6—C5	2.22 (17)	C14—C15—C16—C18	176.71 (12)
O4—C1—C6—C5	178.97 (10)	C19—O8—C18—O9	3.7 (2)
C2—C1—C6—C8	−175.63 (11)	C19—O8—C18—C16	−175.42 (11)
O4—C1—C6—C8	1.11 (17)	C11—C16—C18—O9	26.3 (2)
C9—O2—C8—O3	−1.95 (18)	C15—C16—C18—O9	−151.63 (14)
C9—O2—C8—C6	179.59 (11)	C11—C16—C18—O8	−154.61 (11)
C5—C6—C8—O3	−158.23 (13)	C15—C16—C18—O8	27.48 (16)

### Hydrogen-bond geometry (Å, º)

**Table:**

*D*—H···*A*	*D*—H	H···*A*	*D*···*A*	*D*—H···*A*
C7—H7*B*···O3^i^	0.98	2.52	3.3134 (18)	138
C9—H9*A*···O6^ii^	0.98	2.79	3.3198 (18)	115

Symmetry codes: (i) *x*, *y*+1, *z*; (ii) −*x*, −*y*, −*z*+2.
